# Genomic DNA variation confirmed *Seriola lalandi* comprises three different populations in the Pacific, but with recent divergence

**DOI:** 10.1038/s41598-017-07419-x

**Published:** 2017-08-24

**Authors:** H. K. A. Premachandra, Fabiola Lafarga-De la Cruz, Yutaka Takeuchi, Adam Miller, Stewart Fielder, Wayne O’Connor, Celine H. Frère, Nguyen Hong Nguyen, Ido Bar, Wayne Knibb

**Affiliations:** 1 Genecology Research Centre, Faculty of Science, Health, Education, and Engineering, University of the Sunshine Coast, Maroochydore, DC QLD 4558 Australia; 2 Ensenada Center for Scientific Research and Higher Education (CICESE), Ensenada, B.C. Mexico; 3 Division of Fisheries Resource Sciences, Faculty of Fisheries, Kagoshima University, Shimoarata 4-50-20, Kagoshima city, 890-0056 Japan; 4 Clean Seas Tuna Limited, 7 North Quay Boulevard, Port Lincoln, SA 5606 Australia; 5 Department of Primary Industries, Port Stephens Fisheries Institute, Taylors Beach Road, Taylors Beach, NSW 2316 Australia

## Abstract

Captive breeding programs and aquaculture production have commenced worldwide for the globally distributed yellowtail kingfish (*Seriola lalandi*), and captive bred fingerlings are being shipped from the Southern Hemisphere to be farmed in the Northern Hemisphere. It was recently proposed that Pacific *S*. *lalandi* comprise at least three distinct species that diverged more than 2 million years ago. Here, we tested the hypothesis of different “species” in the Pacific using novel genomic data (namely single nucleotide polymorphisms and diversity array technology markers), as well as mtDNA and DNA microsatellite variation. These new data support the hypothesis of population subdivision between the Northeast Pacific, Northwest Pacific and South Pacific, and genetic divergence indicates restriction to the gene flow between hemispheres. However, our estimates of maximum mtDNA and nuclear DNA divergences of 2.43% and 0.67%, respectively, were within the ranges more commonly observed for populations within species than species within genera. Accordingly our data support the more traditional view that *S*. *lalandi* in the Pacific comprises three distinct populations rather than the subdivisions into several species.

## Introduction

Commercial aquaculture of yellowtail kingfish (*Seriola lalandi* Valenciennes, 1833, Carangidae) has recently commenced around the world. Global aquaculture production of the *Seriola* species exceeds 150,000 tonnes per year^1^, where yellowtail kingfish contributes to the second highest production within the *Seriola* species^2^. At present yellowtail kingfish aquaculture is practised either in sea cages or in land-based facilities and in many different countries including Japan^3^, Australia^4, 5^, New Zealand^6^, USA^7^, Mexico^8^, Chile^9^, Netherlands^10^ and South Africa^11^. A recent study reported the potential expansion to further countries, such as China and Spain^2^. Recent population genetic studies have proposed that globally distributed *S*. *lalandi* is a complex of at least three closely related species with a restriction of gene flow across the equator, and with a divergence of about two millions years^8, 12^. If so, it could be of concern that there is international trade of yellowtail kingfish fingerlings and eggs across the geographical boundaries including across the equator from the Southern to Northern Hemisphere^13, 14^. For example Australian yellowtail kingfish fingerlings have been exported commercially from the Southern Hemisphere to the Northern Hemisphere (Adam Miller, personal communication) and Chilean yellowtail kingfish fingerlings have been exported across the hemispheres to Mexico for commercial aquaculture in 2013^15^. These exchanges or movements of potentially different breeding material have the potential to impact not only local natural wild populations adapted to local regions^16–18^ but also have the potential produce genetic incompatibilities from aquaculture crosses^19, 20^. Indeed, a significant reduction of fitness and viability in F_2_ generation as a consequences of hybrid breakdown of cichlid fish was reported by Stelkens *et al*.^21^, and others reported a significant decrease of fitness with increasing genetic distance^22^ and significant F_2_ hybrid breakdown^23^ in the copepod *Tigriopus californicus*. Given the worldwide distribution of yellowtail kingfish and commercial movement of fingerlings around the world it is important to determine with accuracy the genetic relatedness and consider the possible genetic “compatibility” of different populations around the world.

Most previous population genetic studies of *Seriola* populations applied microsatellite markers and mitochondrial DNA (mtDNA) sequence analysis, or combination of mtDNA and a few nuclear genes for the assessment of genetic diversity and species delimitation^8, 12, 14, 24^. However, the effectiveness of mtDNA and microsatellite loci data in population genetic analysis is questionable due to their low representation of genomic information. Moreover, mtDNA represents only maternal inheritance and may provide a different image of population structure than that provided by biparentally inherited nuclear DNA, as evident in many sea turtle species and white sharks *Carcharodon carcharias* ^25, 26^. While other potential limitations of mtDNA relate to clonal inheritance, neutrality and erratic evolutionary rates suggested that mtDNA may no longer be considered as an ideal marker for evolutionary studies^27^, it still represents a powerful tool for phylogenetic studies, particularly when relating new research to previously published data. The use of DNA microsatellite data has also been challenged for genetic analyses on the grounds of its extremely high mutation rate, size homoplasy, irregularities in mutation process and degradation over time^28, 29^; it is questionable how well evolutionary rates for DNA microsatellite sequences reflect those across the whole genome^30^. Likewise, it is of concern how well just a few nuclear genes reflect whole genome evolution and divergence due to the possibility of locus specific anomalous evolutionary rates^31, 32^. Therefore, to address these concerns arising from the use of traditional markers to resolve phylogenetics, we carried out partial genome sequencing of *S*. *lalandi* from different Pacific regions to reassess the genetic structure of Pacific *S*. *lalandi* populations. This allowed comparison of diversity estimates obtained by traditional methods (mtDNA sequences and DNA microsatellite alleles from new data of this report and previously published mtDNA data) with new estimates, provided for the first time, from partial genomic sequencing.

## Results

### Mitochondrial DNA diversity

Analysis of the mtDNA cytochrome oxidase subunit I (COI) fragments sequenced in this study identified 15 haplotypes containing a total of 28 polymorphic sites (GenBank accession numbers: MF167270 – MF167284; Table 1). From the previously published data, there were 29 haplotypes containing a total of 38 polymorphic sites. The combined new and published data recorded 31 haplotypes containing a total of 41 polymorphic sites (Supplementary Table 1).

**Table 1:** Variable sites of haplotypes based on mtDNA COI sequence.

Variable site				6	9	24	45	51	81	117	126	129	138	225	261	267	288	348	366	384	387	390	459	468	495	498	501	508	516	525	582
Region	Haplo-type	Frequ-ency	GenBank accession no.	T	A	A	T	A	A	A	G	T	C	T	A	G	C	C	A	A	T	T	T	A	A	A	T	T	C	A	G
NW	Hap_1	4.92%	MF167270	.	.	.	.	.	*G*	.	.	.	T	.	.	*A*	.	.	.	**G**	.	*C*	**C**	**T**	G	.	*C*	**C**	**T**	.	*A*
	Hap_2	1.23%	MF167271	.	.	G	.	.	*G*	.	.	.	T	.	.	*A*	.	.	.	**G**	.	*C*	**C**	**T**	G	.	*C*	**C**	**T**	.	*A*
NE	Hap_3	29.92%	MF167272	.	.	.	.	G	.	.	.	.	T	.	G	.	.	T	.	**G**	.	.	**C**	**T**	G	G	.	**C**	**T**	G	.
	Hap_4	9.02%	MF167273	.	.	.	.	G	.	.	.	.	.	.	G	.	.	T	.	**G**	.	.	**C**	**T**	G	G	.	**C**	**T**	G	.
	Hap_5	1.23%	MF167274	.	.	.	.	G	.	.	.	.	T	C	.	.	.	T	.	**G**	.	.	**C**	**T**	.	G	.	**C**	**T**	G	.
	Hap_6	1.64%	MF167275	.	.	.	.	G	.	.	.	.	T	.	.	.	.	T	.	**G**	.	.	**C**	**T**	G	G	.	**C**	**T**	G	.
SP	Hap_7	0.82%	MF167276	.	G	.	.	.	.	G	.	.	.	.	.	.	.	.	.	.	C	.	.	.	.	.	.	.	.	.	.
	Hap_8	44.67%	MF167277	.	G	.	.	.	.	G	.	.	.	.	.	.	.	.	.	.	.	.	.	.	.	.	.	.	.	.	.
	Hap_9	1.64%	MF167278	.	G	.	.	.	.	.	.	.	.	.	.	.	.	.	.	.	.	.	.	.	.	.	.	.	.	.	.
	Hap_10	0.41%	MF167279	.	G	.	.	.	.	G	.	.	.	.	.	.	.	.	G	.	.	.	.	.	.	.	.	.	.	.	.
	Hap_11	2.46%	MF167280	.	.	.	.	.	.	G	.	.	T	.	.	.	.	.	.	.	.	.	.	.	.	.	.	.	.	.	.
	Hap_12	0.41%	MF167281	.	G	.	.	.	.	G	.	C	.	.	.	.	T	.	.	.	.	.	.	.	.	.	.	.	.	.	.
	Hap_13	0.41%	MF167282	.	G	.	.	.	.	G	A	.	.	.	.	.	.	.	.	.	C	.	.	.	.	.	.	.	.	.	.
	Hap_14	0.41%	MF167283	.	G	.	.	.	.	.	.	.	.	.	.	.	.	.	.	.	.	.	.	.	.	.	.	.	.	G	.
	Hap_15	0.82%	MF167284	C	.	.	C	.	.	G	.	.	T	.	.	.	T	.	.	.	.	.	.	.	.	.	.	.	.	.	.

Unique nucleotide positions which separate the Northern and Southern Hemispheres, the Northwest Pacific from the others and the Northeast Pacific from others were represented in bold, italic and underlined text, respectively (NW = Northwest Pacific; NE = Northeast Pacific; SP = South Pacific).

**Figure 1:**
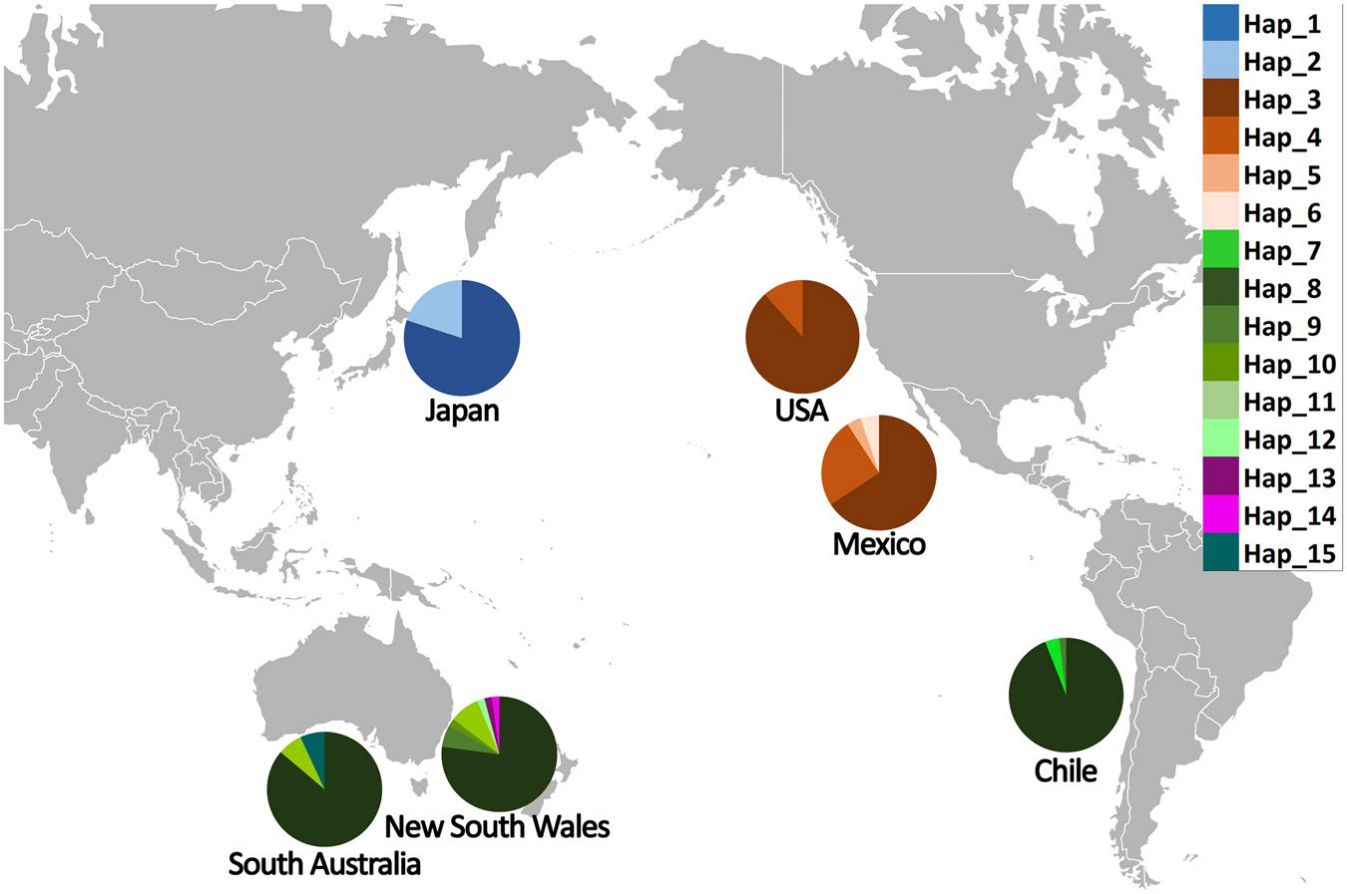
mtDNA haplotype distributions using data of the present study (Hap_1 to Hap_15) among the sample locations. Maps available from Wikipedia Common web page (https://commons.wikimedia.org/wiki/File:Blank_Map_Pacific_World.svg#filelinks) were modified under the Creative Commons public license (https://creativecommons.org/licenses/by-sa/3.0/deed.en) using Adobe Photoshop CS6 software.

There appeared to be marked differences in presence of particular mtDNA haplotypes among the regions of the Southern Hemisphere, Northeast (NE) Pacific and Northwest (NW) Pacific (Fig. 1), but with broad similarities within regions. No haplotypes were shared between regions. Each region (NE Pacific, NW Pacific and South Pacific) shared one major common haplotype and also shared less common haplotypes. Thus one haplotypes were shared between South Pacific locations (Chile, New South Wales: NSW and South Australia: SA); similarly two haplotypes were shared between USA and Mexico (Fig. 1). These general patterns remained when the published data were pooled with data of the present study (Supplementary Fig. 1), except the most common haplotype in South Africa was not the most common haplotype in the other Southern Hemisphere samples.

Multiple sequence alignment confirmed there was no deletions or insertions within the analysed COI sequence fragment. There were five nucleotide positions (represented in bold text, Table 1), in which all Northern Hemisphere samples shared a common nucleotide and which was different from that found in the Southern Hemisphere samples. Similarly there were nucleotide differences at five positions in NW Pacific samples that were categorically different from all others samples (represented in italic text, Table 1); likewise there were three categorical nucleotide differences that separated the NE Pacific from all others (represented in underlined text, Table 1). Again the pooled data present plus published showed the same trends (Supplementary Table 1).

Estimates of genetic diversity from COI (nucleotide diversity: π and haplotype diversity: *h*) indicated relative low values in Chile and higher values in Mexico (Table 2). Analysis of molecular variance (AMOVA) indicated significant population differentiation (F_ST_ = 0.961, *P* < 0.001) among regions where 83% of the total variation were observed among regions (NW Pacific, NE Pacific and South Pacific). Pairwise comparison of F_ST_ indicated significant differences among populations, except among the Australian populations (Table 3). F_ST_ values were highest when comparing between hemispheres, between the NW and NE Pacific locations, but were close to zero when comparing among South Pacific locations. Genetic diversity and AMOVA analyses for the pooled data (present plus published) showed the same trends (Supplementary Table 2 and 3), noting the high diversity in the South African samples.

**Table 2:** Estimates of genetic diversity indices from mtDNA and microsatellite data.

Population	Sample size	mtDNA COI			Microsatellite loci				
		n_h_	π (%)	*h* (±SD)	*g* (±SD)	*N*_a_	*H*_O_	*H*_E_	F_IS_
Japan	15	2	0.060	0.343 ± 0.02	0.800 ± 0.43	9.778	0.726	0.800	0.092
USA	26	2	0.040	0.212 ± 0.09	0.756 ± 0.40	8.778	0.859	0.763	−0.125
Mexico	76	4	0.120	0.507 ± 0.05	0.800 ± 0.42	15.222	0.825	0.816	−0.011
Chile	50	3	0.020	0.117 ± 0.06	0.695 ± 0.37	8.222	0.700	0.706	0.008
NSW	48	7	0.120	0.402 ± 0.08	0.710 ± 0.37	10.333	0.759	0.723	−0.050
SA	29	3	0.150	0.256 ± 0.10	0.692 ± 0.37	8.444	0.719	0.712	−0.010

Abbreviations: Number of haplotypes (n_h_), percent nucleotide diversity (π), haplotype diversity (*h*), gene diversity (*g*), mean number of alleles per locus (*N*_a_), observed heterozygosity (*H*_O_), expected heterozygosity (*H*_E_), inbreeding coefficient (F_IS_), standard deviation (SD). NSW: New South Wales; SA: South Australia.

**Table 3:** Pairwise population matrix of F_ST_ based on mtDNA (below diagonal) and microsatellite (above diagonal) data.

	Japan	USA	Mexico	Chile	NSW	SA
Japan		0.134*	0.094*	0.202*	0.194*	0.204*
USA	0.737*		0.059*	0.227*	0.219*	0.226*
Mexico	0.543*	0.056*		0.200*	0.192*	0.198*
Chile	0.823*	0.849*	0.661*		0.001	0.012*
NSW	0.617*	0.673*	0.539*	0.056*		0.009*
SA	0.711*	0.765*	0.584*	0.025*	0.001	

^*^ *P* < 0.05. NSW: New South Wales; SA: South Australia.

**Figure 2:**
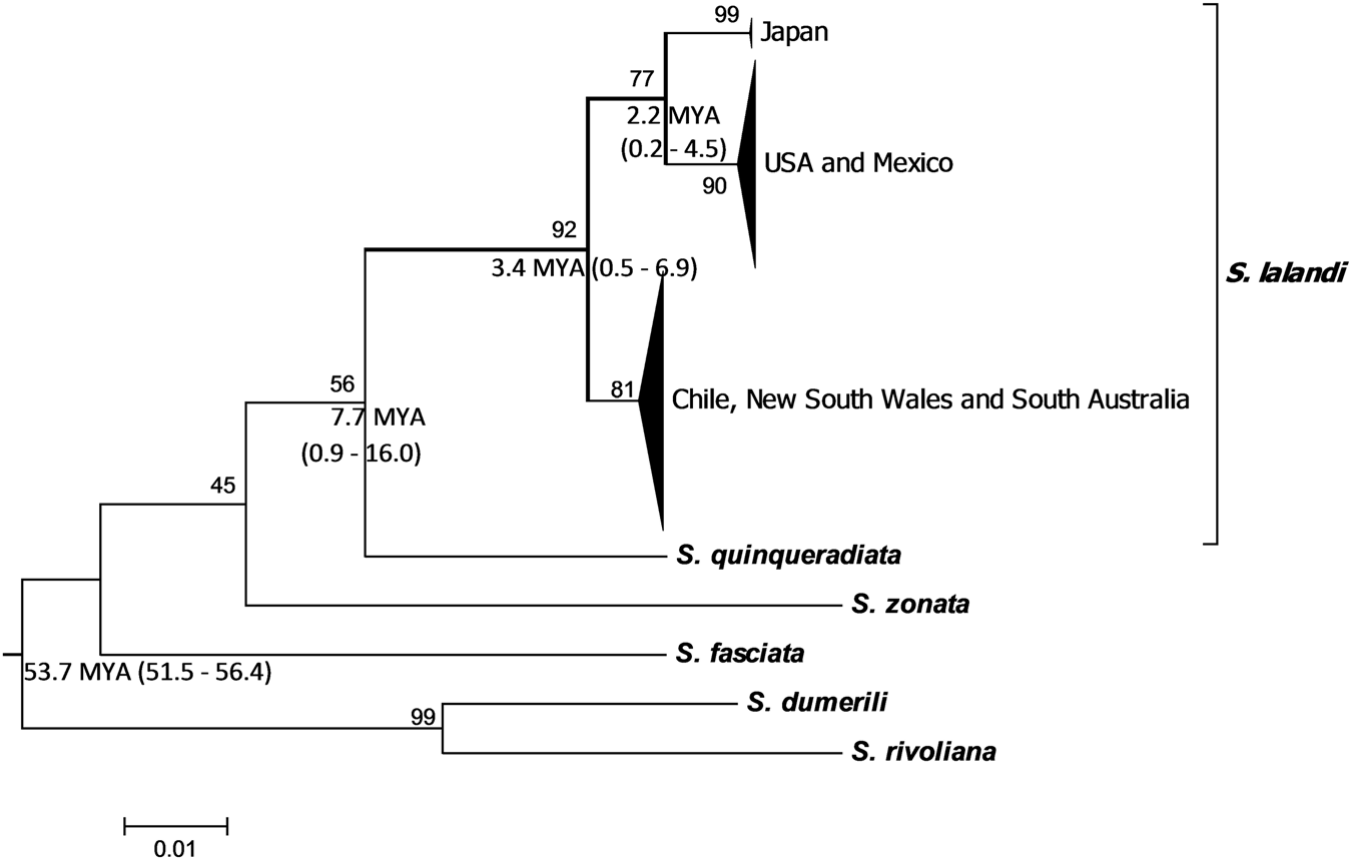
The maximum-likelihood (ML) phylogeny considering mtDNA COI sequences of the *Seriola lalandi* from the present report and sequences from other *Seriola* species. The ML bootstrap percentages are shown above the branches. Mean divergence time estimated from Bayesian inferences in BEAST, with 95% highest posterior density (HPD) for importance nodes are indicated below the nodes.

All phylogenetic tree analyses (Neighbour-Joining, Maximum Parsimony, Maximum Likelihood) using data from the present study formed a single major group of the *S*. *lalandi* sequences, separate from other *Seriola* species, with very high bootstrap support for all the models tested (Fig. 2). However, the single major *S*. *lalandi* group was split into three subgroups corresponding to geographic location (Fig. 2); subgroups were supported with high bootstrap values (>81), where Northern (Japan, USA and Mexico) and Southern (Chile, NSW and SA) Hemisphere locations were separated from each other. Although fish from Japan were placed within the Northern Hemisphere group, they were separated from USA and Mexico (Fig. 2). The same clustering was evident after pooling the published data with the current data (Supplementary Fig. 2), noting that China clustered with Japan and South Africa and New Zealand clustered with Australia and Chile.

Generalized Mixed Yule Coalescent (GMYC) analyses, a likelihood method for delimiting species, indicated that while the Northern and Southern Hemisphere groups have genetic differences, all these lineages, assessed by clade support values, are from a single species group with four clades (single threshold test: null ln *L* = 220.83, max. ln *L* = 229.41, likelihood ratio = 17.16, *P* < 0.001; using pooled data from the current study and published).

Based on molecular dating conducted using Bayesian inferences in BEAST, for the node between Southern and Northern *S*. *lalandi* populations were estimated to be split 3.4 million years ago (MYA) with 95% highest posterior density (HPD) of 0.5–6.9 MYA again using pooled data from the current study and published (Fig. 2 and Supplementary Fig. 3).

Simply considering the percent DNA difference, the maximum COI sequence divergence detected between sampled populations was 2.42% (between Chile and Japan), although samples from the same hemisphere were more closely related (eg. Chile and NSW diverged only by 0.09%, Table 4). The current data of this report and the combined current and published data gave similar results (Supplementary Table 4), noting that the maximum difference for all *S*. *lalandi* samples was 2.43% which is equal to a divergence of 1.2 MYA between hemispheres using the method of Brown *et al*.^33^, whereas the minimum difference between established but related species, eg. *S*. *dumerili* and *S*. *rivoliana* was 6.49%.

**Table 4:** Estimates of pairwise sequence divergence for SNP-based sequences (below diagonal) and mtDNA COI sequences (above diagonal) between populations.

	Japan	USA	Mexico	Chile	NSW	SA
Japan		1.76%	1.78%	2.42%	2.39%	2.42%
USA	na		0.09%	2.38%	2.34%	2.38%
Mexico	0.27%	na		2.33%	2.30%	2.33%
Chile	0.58%	na	0.66%		0.09%	0.01%
NSW	0.57%	na	0.66%	0.22%		0.07%
SA	0.58%	na	0.67%	0.23%	0.23%	

na – data not available. NSW: New South Wales; SA: South Australia.

### Microsatellite DNA variation

Most measures of DNA microsatellite diversity (for example gene diversity, average number of alleles, observed and expected heterozygosity) were broadly similar among the six locations (Table 2). The F_IS_ index did not deviate significantly from zero in any population, suggesting that Hardy-Weinberg equilibrium (HWE) could be assumed in all populations. However, when considering the differences in terms of allele type and frequencies, there were significant differences among populations (AMOVA test F_ST_ = 0.195, *P* < 0.001) and 15% of the molecular variance occurred among populations. Pairwise F_ST_ comparisons between populations varied from 0.001 to 0.227 for microsatellite data and were statistically significant for most pair combinations (*P* < 0.05, Table 3). Higher F_ST_ values were observed between pairs of populations from different hemisphere locations rather than within hemisphere locations.

Analysis of microsatellite data using “STRUCTURE” software indicated the presence of two genetic clusters (K = 2) with a high probability of membership coefficient (Supplementary Fig. 4a). Fish from Northern Hemisphere (Japan, USA and Mexico) were clearly separated from Southern Hemisphere (Chile, NSW and SA) fish populations.

### SNP and DArT markers

A large proportion of the total variation (73%) of single nucleotide polymorphisms (SNPs) was attributed to among regions (South Pacific, Japan, Mexico; AMOVA test, F _ST_ = 0.732, *P* < 0.001), whereas less variation was observed within populations. Pairwise comparison of F_ST_ values also indicated significant differences among hemispheres and regions but not among populations within Southern Hemisphere where values were close to zero (Table 5).

**Table 5:** Pairwise population matrix of F_ST_ analysis of SNPs (below diagonal) and DArT markers (above diagonal) data.

	Japan	Mexico	Chile	NSW	SA
Japan		0.219*	0.272*	0.254*	0.217*
Mexico	0.474*		0.562*	0.456*	0.441*
Chile	0.711*	0.752*		0.017*	0.048*
NSW	0.708*	0.743*	0.006*		0.045*
SA	0.702*	0.741*	0.026*	0.022*	

*P < 0.001. NSW: New South Wales; SA: South Australia.

Principal component analysis (PCA) of SNPs from five sampling locations were sorted into three clusters where Southern Hemisphere locations (Chile, NSW & SA) made one tight cluster and the Mexican population and Japanese population made another two separate clusters, which represent the separation of three regions (Supplementary Fig. 5a). STRUCTURE analysis indicated that the Northern and Southern Hemisphere locations were clearly separated (Supplementary Fig. 4b). DNA divergences for sequence reads were higher (0.57–0.67%) between hemispheres than within hemispheres (0.22–0.27%, Table 4).

AMOVA test for DArT markers (presence/absence of restriction fragments) indicated significant population differentiation (F_ST_ = 0.369, *P* < 0.001) among regions. Pairwise comparison of F_ST_ among populations indicated significant and higher values among the locations from different hemispheres and regions, whereas values were lower (close to zero) among the populations within Southern Hemisphere (Table 5).

PCA of DArT markers indicated presence of two clusters where Southern Hemisphere populations (Chile, NSW & SA) grouped into one cluster and the Mexican and Japanese populations grouped into another (separated by an Eigen vector which contributes to close to 23.5% of the variability in the data; Supplementary Fig. 5b). Similar to the PCA analyses, the STRUCTURE analysis of DArT markers also revealed two clusters (K = 2) with high probability of membership coefficient based on hemisphere locations (Supplementary Fig. 4c).

Three DArT markers (considering either their presence or absence) were present only in Japan and not in other populations, similarly, another three markers distinguished the Mexican population. Conversely, no single South Pacific population had markers restricted only to that population, i.e. all markers from a given South Pacific population were found in each of the other South Pacific populations. When combining the populations in the South Pacific and comparing them to the combined Northern Hemisphere (Japan and Mexico), 20 markers were identified in the Northern Hemisphere which were not present in the South Pacific. Whereas, only two markers were identified in the South Pacific that were not present in the Northern Hemisphere (Supplementary Table 5).

## Discussion

The present mtDNA and DNA microsatellite data indicate the presence of diverged genetic lineages of *S*. *lalandi* from the NW Pacific (Japan and China), the NE Pacific (USA & Mexico), the South Pacific (Chile, NSW & SA) Oceans and South Africa. This interpretation aligns with previous reports of global genetic differences in the *S*. *lalandi* species complex based on mtDNA and microsatellite loci in the NE Pacific, NW Pacific and South Pacific populations^8, 12^. *S*. *lalandi* is known to be a long distance migratory fish: a study on kingfish migration reported that they were able to move >2,000 km from the tagged site, but were recaptured within the same hemispheric locations^34^. Mobility of *S*. *lalandi* was also suggested by genetic differences among years in Chile^35^. However the present data suggest very limited mixing between hemispheres; this may be due to the geographical barriers related to crossing the equator; factors such as the surface gradient of water temperature and salinity that are not preferred by the *S*. *lalandi*, as discussed in previous reports^8, 12^.

Even though the South American and Australian populations are separated by the whole of the Pacific Ocean, there appears to be close similarity among the Southern Hemisphere populations. Purcell *et al*.^8^ report that *S*. *lalandi* from Chile is not significantly different from the *S*. *lalandi* in New Zealand waters. Nugroho *et al*.^24^ and Miller *et al*.^14^ also reported that the *S*. *lalandi* population in Australian waters is not significantly different from that in New Zealand. Martinez-Takeshita *et al*.^12^ considered fish from Chile, New Zealand and South Africa to be one species. Results from the present study revealed that there is no evidence of major genetic differentiation among two Australian populations (NSW & SA), whether considering mtDNA or nuclear DNA data. Furthermore, the current study observed that there is no significant differentiation among the South and East coast Australian, New Zealand and Chilean populations, suggesting *S*. *lalandi* from the South Pacific Ocean is from one genetic lineage. The GMYC analyses did indicate the South African population was a separate genetic lineage from South Pacific samples, even though some haplotypes were shared between South Pacific locations and South Africa. However there were only ten samples available from South Africa, and more samples are needed to confirm the differences between South Africa and other Southern Hemisphere populations.

This picture for the Southern Hemisphere contrasts that from the Northern Hemisphere where mtDNA and nuclear DNA data revealed fish from Japan as a somewhat separate genetic lineage from those in the NE Pacific^8, 12^. Although the geographical distance between Japan (NW Pacific) and USA (NE Pacific) is less than the distance between Australia and Chile, there may be a limitation/barrier to gene flow between NW and NE Pacific locations. Rohde and Hayward^36^ reported that Eastern Pacific Barrier is a 100% effective barrier to dispersal of species of *Scomberomorus*, *Grammatorcynus* and their parasites. Martinez-Takeshita *et al*.^12^ considered the South Pacific offers many island stepping stones for this inshore pelagic species which facilitate migration, stepping stones which are less abundant in the North Pacific. Moreover, Sepulveda and Gonzalez^35^ provided evidance of the migration of genetically different populations over the time and space in South East Pacific Ocean.

Our partial genome sequencing data comprised SNP data and absence/presence of restriction fragments (i.e. the DArT data) were available for Mexico, Japan, Chile and two Australian populations. Broadly, the genome data analysed by a variety of tests supported patterns evident from the mtDNA and DNA microsatellite data, namely major differences between hemispheres but little variation within the Southern Hemisphere. There were some grouping differences regarding Japan which probably reflects the small number of Japanese samples. In general, the SNPs and DArT markers provided larger F_ST_ values than those estimated from the nine microsatellite loci used in the present study and also, the F_ST_ values observed from SNPs were greater than the values observed for DArT markers. Likewise AMOVA analyses for SNPs and DArT data gave much greater F_ST_ estimates, at least two fold of between region variation than those obtained from microsatellite loci. Again, as for F_ST_ values, greater between region variance using AMOVA was evident for SNPs than DArT. A set of DArT markers could be identified that categorically distinguished between the Mexican and Japanese populations and among the Northern and Southern Hemisphere populations.

Recently, Martinez-Takeshita *et al*.^12^ and others^8^ have proposed a revision to the taxonomy of *S*. *lalandi*, specifically that is should be split into three species, namely, resurrection of *Seriola aureovittata* for NW Pacific, *Seriola dorsalis* for NE Pacific, and leaving *S*. *lalandi* as the Southern Hemisphere species. The most straight forward tests for species division are those based just on percent DNA sequence homologies. Considering many different fish species, the recorded average genetic distance between species (within genera) for mtDNA COI gene for marine fish was at least above 9.54%^37, 38^, and at least 5.71% for freshwater species^39^. However, the maximum COI sequence divergence observed between the populations in the present study was just 2.43% (present and published data pooled), which is prima facie evidence that all the populations studied in the present study are from a single species. The maximum nuclear DNA divergence was 0.67%, which is comparable to that between humans and Neanderthals^40^ and there is some debate as to whether humans and Neanderthals are one or separate species^41, 42^. According to the conventional molecular clock of 2% sequence divergence per million years for mtDNA^33, 43^, *S*. *lalandi* populations seem to have diverged about 1.2 MYA between the two hemispheres using our data; this estimate of 1.2 MYA suggests a younger divergence, by about 50%, than previously reported^12^.

More involved tests for species delimitation are available, but do require a greater number of assumptions. Generalized mixed Yule Coalescent analyses indicated all populations belonged to one species group. On the other hand, a divergence time of 0.5 to 6.9 MYA between Hemispheres was estimated from Bayesian inferences in BEAST.

Overall, most of the analyses, both simple and complex point to relatively recent divergence and relatively little genetic difference (apropos that which normally accompanies speciation). The converse does not seem to be the case, that there is consistent and categorical evidence for major genetic change consistent with speciation. For major taxonomic revisions, consistency of the data are probably required and until this evidence is available, the revision proposed by Martinez-Takeshita *et al*.^12^ may be premature.

Nuclear genome sequences were reported to have a lower rate of nucleotide substitution than the mtDNA, about 10-fold lower in primates^33, 44, 45^. However the rate of nucleotide substitution in mtDNA observed in the current study was only about 4 times greater than the nuclear DNA in *S*. *lalandi*. This apparent inconsistency between kingfish and primates indicates a need for more work to properly calibrate the evolutionary rate of kingfish mtDNA sequence divergence over time, and may be needed prior to relying on kingfish mtDNA for major taxonomic revision.

As to whether genetic incompatibilities could arise from mixing Southern and Northern Hemisphere stocks, on one hand our revised estimates of divergence, would lessen this risk as previously considered. On the other hand, our finding using genomic data of many categorical differences could indicate adaption to different local conditions in the different hemispheres, or they could just reflect genetic drift. Perhaps direct empirical testing of F_2_ breakdown would help resolve this matter, and until then any conclusions regarding F_2_ breakdown would only be of a speculative nature.

## Methods and Materials

### Specimen collection and DNA extraction

Fin tissue samples of yellowtail kingfish were collected from six different populations representing New South Wales, Australia (NSW, n = 48), South Australia (SA, n = 29), Chile (n = 50), Japan (n = 15), Mexico (n = 76) and USA (n = 26). Tissue samples were collected into tubes containing 80% ethanol and were stored at 4 °C. Genomic DNA was extracted and purified using the DNeasy Blood and Tissue kits (Qiagen, Hilden, Germany) and the NucleoMag® 96 Tissue kit (MACHEREY-NAGEL GmbH & Co. KG, Germany) following the manufacturers’ protocol and extracted DNA was stored at −18 °C for future analysis.

### Mitochondrial DNA cytochrome oxidase subunit I (COI) gene amplification and sequencing

A length of 655 bp fragment from the COI gene was amplified by polymerase chain reaction (PCR) using gene-specific primers, sense primer FishF1 5′-TCAACCAACCACAAAGACATTGGCAC-3′ and anti-sense primer FishR1 5′-TAGACTTCTGGGTGGCCAAAGAATCA-3′ designed by Ward *et al*.^37^. PCRs were carried out in 25 µL reaction volumes containing 0.5 µL of DNA (50 ng/µL), 12.5 µL of 2× AmpliTaq Gold 360 master mix, 1.0 µL of each gene-specific primer (10 pmol/µL) and 10 µL of PCR-grade H_2_O. Amplifications were performed using an Eppendorf Mastercycler® nexus (Hamburg, Germany). The PCR cycle program consisted of an initial denaturation step at 95 °C for 10 min, followed by 35 cycles of 95 °C for 30 s, 50 °C for 30 s, and 72 °C for 30 s, and a final extension of 72 °C for 10 min. PCR products were subjected to standard DNA sanger sequencing (Macrogen Inc., Korea) from both directions using the gene-specific PCR primer pair mentioned above (FishF1 and FishR1).

### Microsatellite DNA genotyping

Nine established microsatellite markers were used to genotype the 231 specimens. Six markers (Sel001, Sel002, Sel008, Sel011, Sel017 and Sel019) were selected from *S*. *lalandi* (see Supplementary Table 6) transcriptome sequences^5^, and another three loci (Sdu21, Sdu32 and Sdu46) were selected from twenty five published microsatellite primer pairs^46, 47^. Microsatellite primers were combined into two multiplex PCR sets and DNA amplification was achieved using the Qiagen Multiplex PCR PLUS Kit (Qiagen, Germany) in 13.5 µL reactions, each containing 1.25 µL of 10x primer mix, 6.25 µL of Multiplex PCR Master Mix, 2.75 µL of RNase free water, 1.25 µL of Q-Solution and 2.0 µL of approximately 20 ng template DNA. Amplification was performed using an Eppendorf Mastercycler® nexus (Hamburg, Germany) with cycling conditions as follows: initial denaturation at 95 °C for 15 min, followed by 35 cycles of 95 °C for 30 s, 57 °C for 90 s, and 72 °C for 30 s; with a final extension at 68 °C for 30 min. PCR products were separated by capillary electrophoresis on an AB3500 Genetic Analyser (Applied Biosystems) at the University of the Sunshine Coast, Australia.

### DArTseq genotyping

Genomic DNA from five populations were selected for genome-wide markers development; Japan (n = 2), Mexico (n = 38), Chile (n = 20), New South Wales (n = 48) and South Australia (n = 44). DNA aliquots were sent to DArTseq™ (Diversity Arrays Technology Pty. Ltd, Canberra, Australia) for markers development. Four methods of complexity reduction were tested (data not presented) and the *PstI*-*SphI* method was selected. DNA samples were processed in digestion/ligation reactions principally as per Kilian *et al*.^48^ but replacing a single *PstI*-compatible adaptor with two different adaptors corresponding to two different restriction enzyme overhangs. The *PstI*-compatible adapter was designed to include Illumina’s flowcell attachment sequence, sequencing primer sequence and “staggered”, varying length barcode region, similar to the sequence reported by Elshire *et al*.^49^. Reverse adapter contained flowcell attachment region and *SphI*-compatible overhang sequence. Only “mixed fragments” (*PstI*-*SphI*) were effectively amplified in 30 cycles of PCR using the following reaction conditions: an initial denaturation step at 94 °C for 1 min, followed by 30 cycles of 94 °C for 20 s, 58 °C for 30 s, and 72 °C for 45 s; with a final extension at 72 °C for 7 min.

After PCR equimolar amounts of amplification products from each sample of the 96-well microtiter plate were bulked and applied to c-Bot (Illumina) bridge PCR followed by sequencing on an Illumina Hiseq. 2500. The sequencing (single read) was run for 77 cycles, producing 77 bp reads. Sequences generated from each lane were processed using proprietary DArT analytical pipelines. In the primary pipeline the fastq files were first processed to filter away poor quality sequences, applying more stringent selection criteria to the barcode region compared to the rest of the sequence. In that way the assignments of the sequences to specific samples carried in the “barcode split” step were very reliable. Approximately 2,500,000 sequences per barcode/sample were identified and used in marker calling. Finally, identical sequences were collapsed into “fastqcoll files”. The fastqcoll files were “groomed” using DArT PL’s proprietary algorithm which corrects low quality base from singleton tag into a correct base using collapsed tags with multiple members as a template. The “groomed” fastqcoll files were used in the secondary pipeline for DArT PL’s proprietary SNP and silicoDArT (presence/absence of restriction fragments in representation; DArT markers) calling algorithms (DArTsoft14). For SNP calling all tags from all libraries included in the DArTsoft14 analysis are clustered using DArT PL’s C++ algorithm at the threshold distance of 3, followed by parsing of the clusters into separate SNP loci using a range of technical parameters, especially the balance of read counts for the allelic pairs. Additional selection criteria were added to the algorithm based on analysis of approximately 1,000 controlled cross populations. Testing for Mendelian distribution of alleles in these populations facilitated selection of technical parameters discriminating well true allelic variants from paralogous sequences. In addition multiple samples were processed from DNA to allelic calls as technical replicates and scoring consistency was used as the main selection criteria for high quality/low error rate markers. Calling quality was assured by high average read depth per locus (Average across all markers was over 30 reads/locus).

### Data analysis

#### Mitochondrial DNA COI fragment

Two hundred and forty-four mtDNA sequences generated in this study were trimmed to 604 bp (see Table 1 for GenBank accession numbers) and were analysed using the BLAST algorithm from NCBI (https://blast.ncbi.nlm.nih.gov/Blast.cgi). COI region sequences were evaluated and aligned using ClustalW multiple sequence alignment program in BioEdit sequence alignment editor V.7.2.5^50^. Published sequences were also collated by directly acquiring homologous sequences from GenBank (Genbank accession numbers: EF609460^38^; HM007727-HM007730^51^; JF494499; JF494500; JQ738432; JQ738434; KM877615-KM877656^12^; KU312946-KU312964^35^; KX781877); sequences were trimmed to 604 bp to correspond to the sequences generated in the present study. Also we regenerated DNA sequences from published haplotype frequencies as found in Sepúlveda and González^35^; for example, the sequence of the “H1” haplotype was reported along with its frequency (which was 80%) from a total of 291; thus we inferred there were a total count of 233 “H1” haplotypes.

Analysis of molecular variance (AMOVA) test and pairwise genetic differences (F_ST_) were estimated as indicators of genetic differentiation between all samples groups using Arlequin 3.5.2 and significance was assessed using 1,000 permutations^52^. DNA polymorphism/divergence analysis were conducted to estimate the average number of nucleotide differences (k), nucleotide diversity (π) and haplotype diversity (*h*) using DnaSP v. 5.0^53^.

Prior to the construction of phylogenetic trees, an assessment was made using MEGA 7.0^54^ to find the best model of sequence evolution. The best model for COI was determined to be the “K2 + G: Kimura 2-parameter + Gamma” model which was selected based on Akaike Information Criterion (AIC) and Bayesian Information Criterion (BIC) values^55^. Phylogenetic tree analyses were conducted with aligned sequences from the present study (244 sequences) and previously published (341 sequences) COI gene sequences from *S*. *lalandi* and from related *Seriola* species as outgroups (*S*. *dumerili*: KC501452; *S*. *rivoliana*: JN021317; *S*. *zonata*: KF930431; *S*. *fasciata*: KF930429; *S*. *quinqueradiata*: KU168712) using Neighbour-Joining (NJ)^56^, Maximum Parsimony (MP)^57^ and Maximum Likelihood (ML)^58^ methods, with 1,000 bootstrap replicates in MEGA 7.0^54^.

To test how many different species were evident from the COI sequences, a species delimitation analysis was carried out using the Generalized Mixed Yule Coalescent (GMYC) approach^59^. Bayesian phylogenetic inferences were characterized with HKY + G (Hasegawa-Kishino-Yano + Gamma) model, assuming coalescent constant population priors with relaxed lognormal clock^60^ in BEAST v2.4.6^61^. Single representatives of given COI haplotypes sequences (to avoid many zero-length branches) together with five outgroup sequences (from different *Seriola* species used in ML analysis) were used in phylogeny analysis. BEAST was run for 10 million of Markov-Chain Monte Carlo (MCMC) chain length (sampled every 1000 generations; burn-in 10%), which reached convergence with effective sample size (ESS) > 200 for all parameters. Independent GMYC analyses for the trees generated from BEAST were conducted using “rncl”^62^ and “splits”^59^ packages in R^63^ to discover species.

Divergence time estimates were assessed using two approaches. First, molecular dating for COI haplotype sequences were conducted using Bayesian inferences in BEAST v2.4.6^61^, setting birth-death tree prior, since this is well suited and used for multi species data with varying degrees of lineage divergence^64^. The uncorrelated log-normal relaxed molecular clock was selected for node ages estimates. Monophyly constraints were enforced for all clades represented by calibration points. Calibration points were set to 55.0 MYA (SD = 1.0) to calibrate the ancestral node of the genus *Seriola* from the fossil record^65, 66^ that has already been used in time-calibration studies^67^ and 52.0 MYA (SD = 1.0) for the split between *Seriolina nigrofasciata* and *Elagatis bipinnulata* derived from phylogenetic data^68^ and TIMETREE database^69, 70^ and thus here included *S*. *nigrofasciata* and *E*. *bipinnulata* (GenBank accession No. HQ560985 and JF493409, respectively) as outgroups. Three independent runs were implemented with 10 million MCMC steps and sampled every 1000 generations. A burn-in of 10% was used and the convergence of all parameters was assessed (ESS > 200) using the TRACER v1.6^71^. Subsequently, TreeAnotator v2.4.6^61^ was used to generate the consensus tree with median node age and FigTree v1.4.3^72^ was used to visualise the annotated tree.

The second approach to estimate the divergence time among populations was to calculate pairwise sequence divergence percentages (1-sequence identity) using BioEdit v7.2.5^50^. The number of base differences per site was averaged over all corresponding sequence pairs for the full COI data set and calibrated according to Brown *et al*.^33^.

#### Microsatellite DNA markers

Molecular diversity indices including gene diversity (*g*), mean number of alleles per locus (*N*_a_), observed heterozygosity (*H*_O_) and expected heterozygosity (*H*_E_) were estimated with 10,000 permutations and Hardy-Weinberg equilibrium (HWE) tests were performed with 1,000,000 Markov Chain steps and 100,000 dememorization steps using Arlequin V. 3.5.2^52^. AMOVA test and population level of inbreeding (F_IS_) were performed using Genetic Analysis in Excel (GenAlEx) V. 6.5^73, 74^. Microsatellite data were used to conduct Bayesian cluster analysis using STRUCTURE software V. 2.3 program^75^, assessing individuals to genetic clusters without a priori population definition and using ‘admixture’ ancestry model with correlated allele frequencies. The number of assumed genetic clusters (K) was set to vary between 1 to 6 and the program setup for five independent runs with burn-in period of 100,000 iterations and 100,000 MCMC (Markov Chain Monte Carlo) iterations. The most likely number of K was determined using the STRUCTURE HARVESTER program^76^.

#### SNP and DArT markers

SNP and DArT markers (presence/absence data) data were filtered to remove any loci which had missing marker information for more than 5% of the samples within each population, as well as those with multiple markers in the same locus, to allow assumption of HWE and single-loci markers in downstream population structure and sequence homology analyses. In addition, samples with missing data in more than 100 markers were filtered out. Markers unique to each population were determined as those present in at least 95% of the individuals in the population, while present in not more than 5% of the individuals of all other populations. Data filtration and analysis was performed in the R statistical programing language v3.2.1^63^ environment, using the ‘adegenet’ package^77^.

Both DArT and SNPs data were subjected to AMOVA test using Genetic Analysis in Excel (GenAlEx) V. 6.5^73, 74^ and Arlequin V. 3.5.2^52^ for population differentiation. In addition, a Bayesian model-based cluster analysis was performed for three independent runs with K from 1 to 5 and each run with a burn-in period of 100,000 iterations followed by 100,000 MCMC iterations, assuming an admixture ancestry model and correlated allele frequencies using STRUCTURE software V. 2.3 program^75^. The results of STRUCTURE were subsequently analysed to determine the most likely number of K using the STRUCTURE HARVESTER program^76^.

Since each single-loci DArTseq™ marker comprised of a single polymorphic base (SNP) in a consensus sequence of fixed tag length (70 bp in this case), the sequence homology (in percentage) across one allele of a polymorphic marker can be defined as (1–1/70 bp) × 100. Therefore, the overall sequence dissimilarity between samples could be calculated as the total number of varying SNPs (S_v_) across both alleles of single-loci markers, divided by the total number of alleles in the markers (2n) multiplied by the tag length (l), as presented in Equation 1. The overall sequence homology within and between populations was then calculated as the mean of homologies from each pairwise comparison of individual samples from a single or compared populations, respectively.

Equation 1. SNP-based sequence homology calculation

$${H}_{s}=1-\,\frac{{\sum }_{i=0}^{2n}\,{s}_{v}}{2n\times l}$$

## Electronic supplementary material

Genomic DNA variation confirmed Seriola lalandi comprises three different populations in the Pacific, but with recent divergence
