# Supplementary material for: Genomic DNA variation confirmed Seriola lalandi comprises three different populations in the Pacific, but with recent divergence
Source: Sci Rep. 2017 Aug 24;7:9386. doi: 10.1038/s41598-017-07419-x (PMC5571200; doi:10.1038/s41598-017-07419-x)
Supplement: Supplementary file 1 — Genomic DNA variation confirmed Seriola lalandi comprises three different populations in the Pacific, but with recent divergence [file 41598_2017_7419_MOESM1_ESM.pdf]

Supplementary information

Genomic DNA variation confirmed *Seriola lalandi* is three different populations in the Pacific, but with recent divergence

H.K.A. Premachandra[a], Fabiola Lafarga-De la Cruz[b], Yutaka Takeuchi[c], Adam Miller[d], Stewart Fielder[e], Wayne O'Connor[e], Celine H. Frère[a], Nguyen Hong Nguyen[a], Ido Bar[a], Wayne Knibb[a, *]

Supplementary Table 1. All the variable sites of haplotypes based on published and present mtDNA COI sequences. Unique nucleotide positions which separate the Northern and Southern Hemisphere, the Northwest Pacific from others and Northeast Pacific from others were shaded in black, dark grey and light grey, respectively.

		Variable site	6	9	24	27	39	45	51	74	81	84	117	126	129	135	138	186	225	261	267	276	288	294	315	318	348	366	384	387	390	447	459	468	469	489	495	498	501	508	516	525	582
Haplotype	Frequency	Country/Region	T	G	A	G	T	T	A	T	A	G	G	G	T	G	C	C	T	A	G	G	C	G	T	C	C	A	A	T	T	T	T	A	C	G	A	A	T	T	C	A	G
Hap_1	3.59%	Japan, China	.	A	.	.	.	.	.	.	G	.	A	.	.	.	T	.	.	.	A	.	.	.	.	.	.	.	G	.	C	.	C	T	.	.	G	.	C	C	T	.	A
Hap_2	0.85%	Japan	.	A	G	.	.	.	.	.	G	.	A	.	.	.	T	.	.	.	A	.	.	.	.	.	.	.	G	.	C	.	C	T	.	.	G	.	C	C	T	.	A
Hap_3	0.17%	Japan	.	A	.	.	.	.	.	.	G	.	A	.	.	.	T	.	.	.	A	.	.	.	.	T	.	.	G	.	C	.	C	T	.	.	G	.	C	C	T	.	A
Hap_4	14.87%	USA, Mexico	.	A	.	.	.	.	G	.	.	.	A	.	.	.	T	.	.	G	.	.	.	.	.	.	T	.	G	.	.	.	C	T	.	.	G	G	.	C	T	G	.
Hap_5	3.93%	USA, Mexico	.	A	.	.	.	.	G	.	.	.	A	.	.	.	.	.	.	G	.	.	.	.	.	.	T	.	G	.	.	.	C	T	.	.	G	G	.	C	T	G	.
Hap_6	1.20%	USA, Mexico	.	A	.	.	.	.	G	.	.	.	A	.	.	.	T	.	C	.	.	.	.	.	.	.	T	.	G	.	.	.	C	T	.	.	.	G	.	C	T	G	.
Hap_7	0.85%	Mexico	.	A	.	.	.	.	G	.	.	.	A	.	.	.	T	.	.	.	.	.	.	.	.	.	T	.	G	.	.	.	C	T	.	.	G	G	.	C	T	G	.
Hap_8	0.17%	Mexico	.	A	.	.	.	.	G	.	.	.	A	.	.	.	T	.	.	G	.	A	.	.	.	.	T	.	G	.	.	.	C	T	.	.	G	G	.	C	T	G	.
Hap_9	0.17%	Mexico	.	A	.	.	.	.	G	.	.	.	A	.	.	.	T	.	.	G	.	.	.	.	.	.	T	.	G	.	.	.	C	T	.	.	C	G	.	C	T	G	.
Hap_10	0.85%	Chile	.	.	.	.	.	.	.	.	.	.	.	.	.	.	.	.	.	.	.	.	.	.	.	.	.	.	.	C	.	.	.	.	.	.	.	.	.	.	.	.	.
Hap_11	61.71%	Chile, NSW, SA, NZ	.	.	.	.	.	.	.	.	.	.	.	.	.	.	.	.	.	.	.	.	.	.	.	.	.	.	.	.	.	.	.	.	.	.	.	.	.	.	.	.	.
Hap_12	3.59%	Chile, NSW	.	.	.	.	.	.	.	.	.	.	A	.	.	.	.	.	.	.	.	.	.	.	.	.	.	.	.	.	.	.	.	.	.	.	.	.	.	.	.	.	.
Hap_13	0.85%	Chile	.	.	.	.	.	.	.	.	.	.	.	.	.	.	.	.	.	.	.	.	.	A	.	.	.	.	.	.	.	.	.	.	.	.	.	.	.	.	.	.	.
Hap_14	2.91%	Chile, NSW, SA, S_Af	.	A	.	.	.	.	.	.	.	.	.	.	.	.	T	.	.	.	.	.	.	.	.	.	.	.	.	.	.	.	.	.	.	.	.	.	.	.	.	.	.
Hap_15	0.34%	Chile	.	.	.	.	.	.	.	.	.	.	.	.	.	.	.	.	.	.	.	.	.	.	.	.	.	.	.	.	.	.	.	.	.	.	.	.	C	.	.	.	.
Hap_16	0.34%	Chile, NSW	.	.	.	.	.	.	.	.	.	.	A	.	.	.	.	.	.	.	.	.	.	.	.	.	.	.	.	.	.	.	.	.	.	.	.	.	.	.	.	G	.
Hap_17	0.17%	Chile	.	.	.	.	.	.	.	.	.	.	.	.	.	.	.	.	.	.	.	.	.	.	.	.	.	.	.	.	.	.	.	.	.	A	.	.	.	.	.	.	.
Hap_18	0.17%	Chile	.	.	.	.	.	.	.	.	.	.	.	.	.	A	.	.	.	.	.	.	.	.	.	.	.	.	.	.	.	.	.	.	.	.	.	.	.	.	.	.	.
Hap_19	0.17%	Chile	.	.	.	.	.	.	.	.	.	.	.	.	.	.	.	T	.	.	.	.	.	.	.	.	.	.	.	.	.	.	.	.	.	.	.	.	.	.	.	.	.
Hap_20	0.17%	Chile	.	.	.	.	.	.	.	G	.	.	.	.	.	.	.	.	.	.	.	.	.	.	.	.	.	.	.	.	.	.	.	.	.	.	.	.	.	.	.	.	.
Hap_21	0.68%	Chile, NSW	.	.	.	.	.	.	.	.	.	.	.	.	.	.	.	.	.	.	.	.	.	.	.	.	.	G	.	.	.	.	.	.	.	.	.	.	.	.	.	.	.
Hap_22	0.17%	Chile	.	.	.	A	.	.	.	.	.	.	.	.	.	.	.	.	.	.	.	.	.	.	.	.	.	.	.	.	.	.	.	.	.	.	.	.	.	.	.	.	.
Hap_23	0.17%	Chile	.	.	.	.	.	.	.	.	.	.	.	.	.	.	.	.	.	.	.	.	.	.	.	.	.	.	.	.	.	.	.	.	G	.	.	.	.	.	.	.	.
Hap_24	0.17%	Chile	.	.	.	.	.	.	.	.	.	.	.	.	.	.	T	.	.	.	.	.	.	.	.	.	.	.	.	.	.	.	.	.	.	.	.	.	.	.	.	.	.
Hap_25	0.34%	Chile, NSW	.	.	.	.	.	.	.	.	.	.	.	.	C	.	.	.	.	.	.	.	T	.	.	.	.	.	.	.	.	.	.	.	.	.	.	.	.	.	.	.	.
Hap_26	0.17%	Chile	.	.	.	.	.	.	.	.	.	.	.	.	.	.	.	.	.	.	.	.	.	.	C	.	.	.	.	.	.	.	.	.	.	.	.	.	.	.	.	.	.
Hap_27	0.17%	NSW	.	.	.	.	.	.	.	.	.	.	.	A	.	.	.	.	.	.	.	.	.	.	.	.	.	.	.	C	.	.	.	.	.	.	.	.	.	.	.	.	.
Hap_28	0.34%	SA	C	A	.	.	.	C	.	.	.	.	.	.	.	.	T	.	.	.	.	.	T	.	.	.	.	.	.	.	.	.	.	.	.	.	.	.	.	.	.	.	.
Hap_29	0.34%	South Africa	.	A	.	.	.	.	.	.	.	.	.	.	.	.	T	.	.	G	.	.	.	.	.	.	.	.	.	.	.	.	.	.	.	.	.	.	.	.	.	.	.
Hap_30	0.17%	South Africa	.	A	.	.	C	.	.	.	.	.	.	.	.	.	T	.	.	G	.	.	.	.	.	.	.	.	.	.	.	.	.	.	.	.	.	.	.	.	.	.	.
Hap_31	0.17%	South Africa	.	A	.	.	.	.	.	.	.	A	.	.	.	.	T	.	.	.	.	.	.	.	.	.	.	.	.	.	.	C	.	.	.	.	.	.	.	.	.	.	.

Abbreviations: New South Wales (NSW); South Australia (SA); New Zealand (NZ); South Africa (S_Af).

Haplotype numbers (column 1) are generated by Arlequin software analyses for this particular Table and do not correspond to those haplotype names given elsewhere.

Supplementary Table 2. Estimates of genetic diversity indices based on published and present mtDNA COI sequences.

Population	mtDNA COI			
	n	n_h	π (%)	*h* (±SD)
Japan	25	3	0.068	0.397 ± 0.10
China	2	1	0.000	0.000 ± 0.00
USA	30	3	0.064	0.246 ± 0.09
Mexico	94	6	0.137	0.532 ± 0.05
Chile	343	17	0.052	0.257 ± 0.07
NSW	50	7	0.114	0.388 ± 0.08
SA	29	3	0.148	0.256 ± 0.10
NZ	2	1	0.000	0.000 ± 0.00
S_Africa	10	4	0.177	0.644 ± 0.15

Abbreviations: Sample size (n), number of haplotypes (n_h), nucleotide diversity (π), haplotype diversity (*h*), standard deviation (SD), New South Wales (NSW), South Australia (SA), New Zealand (NZ), South Africa (S_Africa).

Supplementary Table 3. Pairwise population matrix of F_{ST} based on published and present mtDNA COI sequences.

	Japan	China	USA	Mexico	Chile	NSW	SA	NZ
Japan								
China	-0.162							
USA	0.684*	0.775*						
Mexico	0.514*	0.534*	0.045*					
Chile	0.717*	0.759*	0.745*	0.657*				
NSW	0.609*	0.652*	0.670*	0.528*	0.011			
SA	0.677*	0.766*	0.749*	0.563*	0.003	-0.002		
NZ	0.650*	1.000	0.775*	0.534*	-0.260	-0.221	-0.265	
S_Africa	0.512*	0.480*	0.625*	0.435*	0.511*	0.510*	0.598*	0.480*

* $P < 0.05$.

Abbreviations: New South Wales (NSW), South Australia (SA), New Zealand (NZ), South Africa (S_Africa).

Supplementary Table 4. Estimates of pairwise sequence divergence based on published and present mtDNA COI sequences between populations and other *Seriola* species (all values are in percentages).

	China	USA	Mexico	Chile	NSW	SA	NZ	S_Africa	*S. dumerili*	*S. rivoliana*	*S. zonata*	*S. fasciata*	*S. quinqueradiata*
Japan	0.05	1.77	1.79	2.43	2.40	2.41	2.42	2.16	9.47	10.13	4.57	10.02	5.72
China		1.73	1.75	2.39	2.37	2.38	2.40	2.12	9.43	10.10	4.53	10.00	5.70
USA			0.11	2.36	2.34	2.36	2.37	2.04	9.80	11.41	4.54	9.69	6.33
Mexico				2.33	2.31	2.33	2.34	2.05	9.80	11.41	4.54	9.68	6.35
Chile					0.10	0.12	0.03	0.49	8.94	10.54	3.37	9.20	5.51
NSW						0.15	0.07	0.48	8.93	10.53	3.36	9.18	5.50
SA							0.09	0.46	8.88	10.48	3.34	9.18	5.49
NZ								0.48	8.93	10.53	3.37	9.20	5.50
S_Africa									8.66	10.28	3.10	8.88	5.28
S. dumerili										6.49	9.43	11.30	9.70
S. rivoliana											11.16	11.17	10.23
S. zonata												9.40	6.50
S. fasciata													9.20

Abbreviations: New South Wales (NSW), South Australia (SA), New Zealand (NZ), South Africa (S_Africa).

Supplementary Table 5. Diversity array (DArT) markers unique for each population and hemispheres (NH = Northern Hemisphere; SP = South Pacific).

DArT_tag	Tag sequence is unique to these regions	Tag_sequence	Mean of the presence/absence (1/0) of tags in populations				
			Japan	Mexico	Chile	NSW	SA
13781242	Japan	TGCAGAGTGTTCCTCCTCCTCCTCCTCCTCCTCAGGAACTCTGTATATCCTAAAGACAGTATGGTTGAG	1	0	0	0	0
13783891	Japan	TGCAGGTAATTCAAAAATATAAGTTCTTTTTTTGAATGCATGAGATCGGAAGAGCGGTTCAGCAGGAAT	1	0	0	0	0
13782960	Japan	TGCAGCATAAGGCTCCCATATCATGTCCTGCAATTCATTATGAGCGCTGTAGTATCATGTGTGTCTGTG	1	0	0	0.021	0
13792414	Mexico	TGCAGAGCTTTTTCTCCACTTCTGACAGAGCCCAGTCCAAATCCTCATGGTGATCAGATGTGTCCCTTG	0	0.973	0	0	0
13799763	Mexico	TGCAGTGGACATATCAGTTCAGTTATGCATGAGATCGGAAGAGCGGTTCAGCAGGAATGCCGAGACCGA	0	1	0	0	0
13788627	Mexico	TGCAGCCCAGCAGTTGCTGTACTAATGTCAGCATGAGATCGGAAGAGCGGTTCAGCAGGAATGCCGAGA	1	0	1	1	1
13800576	NH	TGCAGCGTGCATGTGTGTGTGTGTGCGCACATCCACACGTTTGTGCATTTGTGCATGAGATCGGAAGAG	1	1	0	0.044	0
13792462	NH	TGCAGTGAAGTTCTTGAAGAGTACACACAGAAGAGACACAATCTTTCCTAACTCACTTCTTCCTAACAC	1	1	0	0.022	0
13780593	NH	TGCAGTGAGTCGCTATCAGAAAGTGACAAGGTGGGCCGGGCTAGCGGGTCAGCATGAGATCGGAAGAGC	1	1	0	0	0
13801331	NH	TGCAGTCTAGAGTGCAGCTGCACACCCTCCAACAGATGAGCTGTGTGTGTGTGTGTGTGTGTGTGTGTG	1	1	0	0	0
13781048	NH	TGCAGTGTCTCACACACACACACACACATGCGCACGCATGAGATCGGAAGAGCGGTTCAGCAGGAATGC	1	1	0	0	0
13781029	NH	TGCAGTTTTTTATAATGCAACCTACCATACAAAATAATGTAGAAATGGCCTGCTCCTTGGCCTTGGAAG	1	1	0	0	0
13789043	NH	TGCAGCCCTAACTTCAGCTTGCAGTACAAATATTATAATTATGCATGAGATCGGAAGAGCGGTTCAGCA	1	1	0	0	0
13780998	NH	TGCAGAAAACACACTTTGAGGATGACGACTGCTAACAACACACAAAGACACACAGAGACTGCTATGTTT	1	1	0	0	0
13799426	NH	TGCAGCGAGAAGTTGTCAAATAACAAAACAAGCAATGTGGAAAGACATTTCCAGGGAAGGCATGAGATC	1	1	0	0	0
13799781	NH	TGCAGACCAAATCCCAGGCATGAGATCGGAAGAGCGGTTCAGCAGGAATGCCGAGACCGATCTCGTATG	1	1	0	0	0
13802148	NH	TGCAGGAGTGAAACGGCCGGTTTGGAGTCTGTCGTGAACATAGAGCAGTGGGCAAAAGAGTTTTAGGGA	1	1	0	0	0
20270489	NH	TGCAGCGGCAACATCTCCACACAGGGATGTACCCGTCGTCATGATGGCTCACACTGGTACTGCCTCTTC	1	1	0	0	0
20270139	NH	TGCAGGCCACAAACACACAAACACACACACACACACACAGACACGCAGCCTGTGCAGAGGTGGCACTAC	1	0.973	0	0	0
13792461	NH	TGCAGCGCGTCTGGGGAGAGAGCTGTTTGACAATAAACCCAGCATACACTGCACATGCAGCCGCTGTGT	1	1	0	0	0
13781010	NH	TGCAGCTGACTGACAGCTCACAGGAATAAGGCTTTTTCCTGGTTTCCGTCCTCCTTGAAACCCATCCAT	1	0.974	0	0	0
13793289	NH	TGCAGTCTGGGGACTCGATGTGCATGTGAGAGGGTTTGCATGAGATCGGAAGAGCGGTTCAGCAGGAAT	1	1	0	0	0
13793383	NH	TGCAGCTTTTACAGAAAACTTCAATGAAAAGTATTCACTACAAGACAGAGGTGATGTTTTATAATATCA	1	1	0	0	0
13788043	NH	TGCAGCTACGTATATTGATGAGGGACAGCAACAATGAACTTAAGTCAAAATATCTGCATTTTCTATGTA	1	1	0	0	0
13781282	NH	TGCAGTGTCTCACACACACACACACATGCGCACGCATGAGATCGGAAGAGCGGTTCAGCAGGAATGCCG	1	1	0	0	0
20269361	NH	TGCAGTGTACGTTAACGTACAGAGCTGCATCATTTCCAATGTAATTGATGCTCACACACATTTGTTTCT	1	1	0	0	0
13788845	SP	TGCAGTGAGTGCCGCATGTCCTCTTGAGCATGAGATCGGAAGAGCGGTTCAGCAGGAATGCCGAGACCG	0	0	1	0.957	1
13792781	SP	TGCAGCAAACTGAAGGCGAAGTTGAATTTTGCATGAGATCGGAAGAGCGGTTCAGCAGGAATGCCGAGA	0	0	1	1	1

Supplementary Table 6. PCR primers used for microsatellite genotyping.

Primer	Forward sequence (5´-3´)	Reverse sequence (5´-3´)
Sel001	GAGCTGTGGAACAGGAAACC	CATCCTGGGTACAGGAGCAG
Sel002	ATCAGAAATGGACCGAGGTG	TGGTCCTCCGAGAAAGACTC
Sel008	TGTGGGGAACTGTGTTTTCA	AGAGGACAGAGCCAACTTGC
Sel011	TTCAAGAGTCATCAACATCATCG	GCAAAGCTGAAAGGCAAAGT
Sel017	CAGGGCAGAGGGAGAAGTTA	GGTTACCAGGACGGCTATCA
Sel019	CTATGGCCTCATTCCAGTGC	AAGTACCACAGTGCCCAAGG

Primers Sel001 – Sel019. Methods to isolate primers are given in Whatmore *et al.*[1].

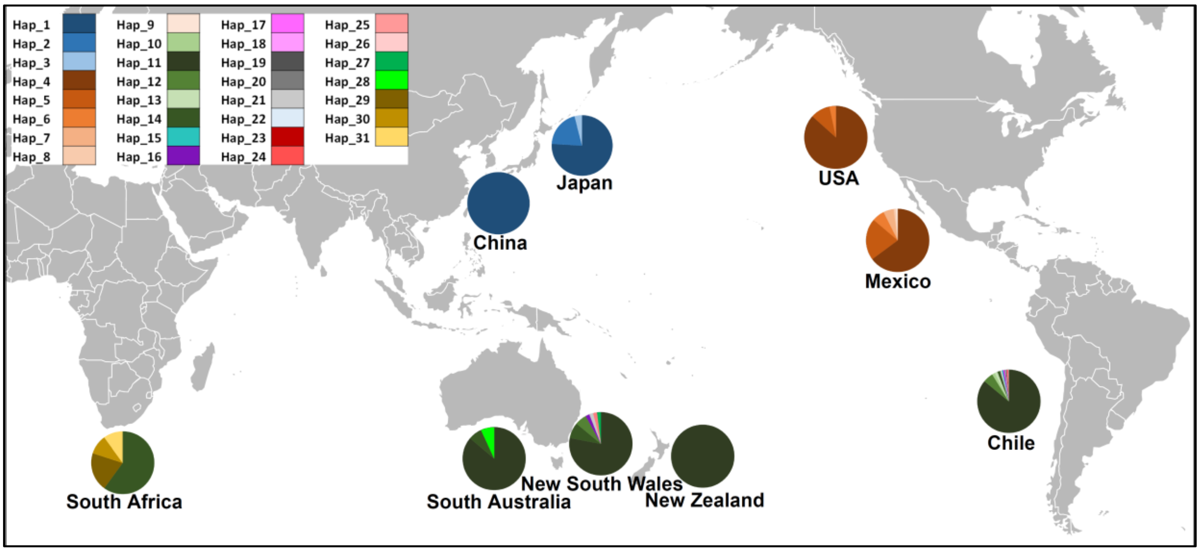

Supplementary Figure 1. mtDNA haplotype distributions using data of the present study pooled with published data . Maps available from Wikipedia Common web page (https://commons.wikimedia.org/wiki/File:Blank_Map_Pacific_World.svg#filelinks) were modified under the Creative Commons public license (https://creativecommons.org/licenses/by-sa/3.0/deed.en) using Adobe Photoshop CS6 software.

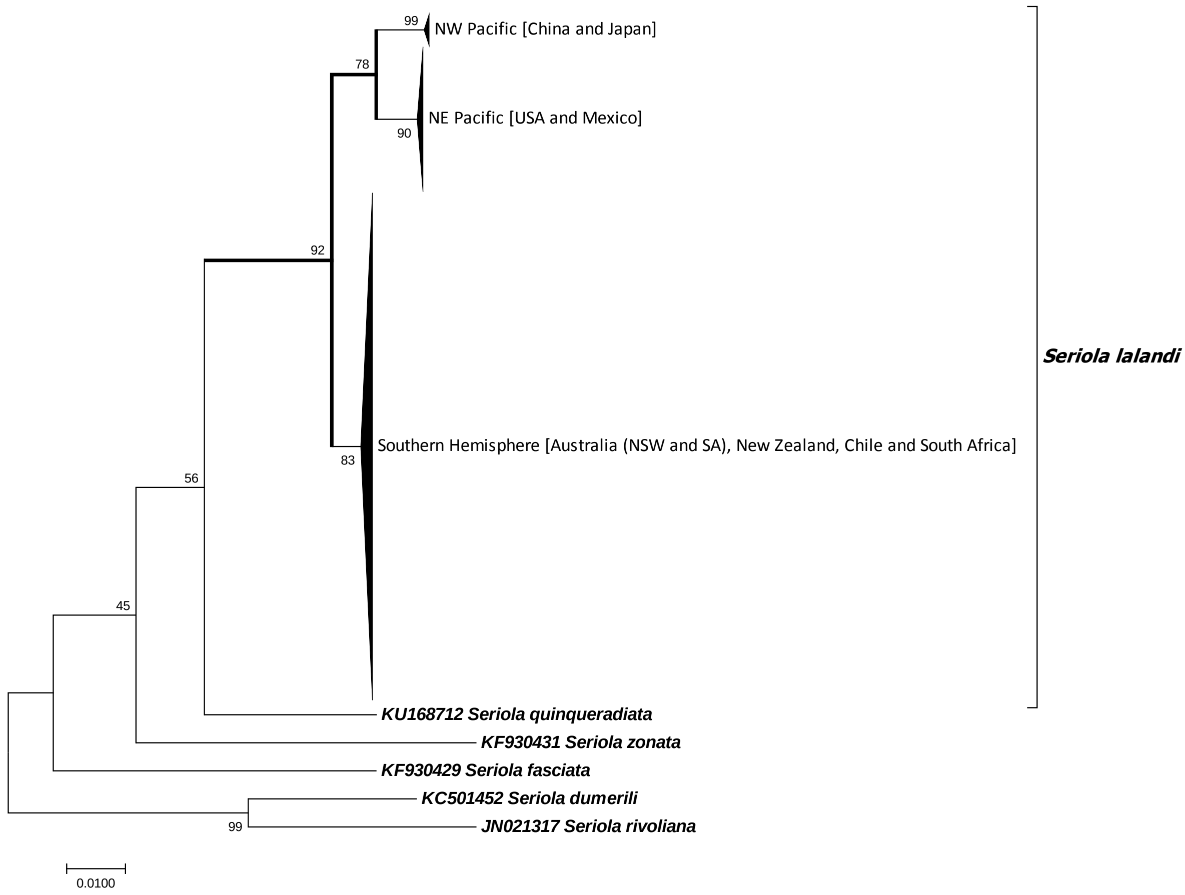

Supplementary Figure 2. The maximum-likelihood (ML) phylogeny considering mtDNA COI sequences from the present report, published sequences and sequences from other *Seriola* species. The ML bootstrap percentages are shown above the branches and GenBank accession numbers for related species are shown with taxa label.

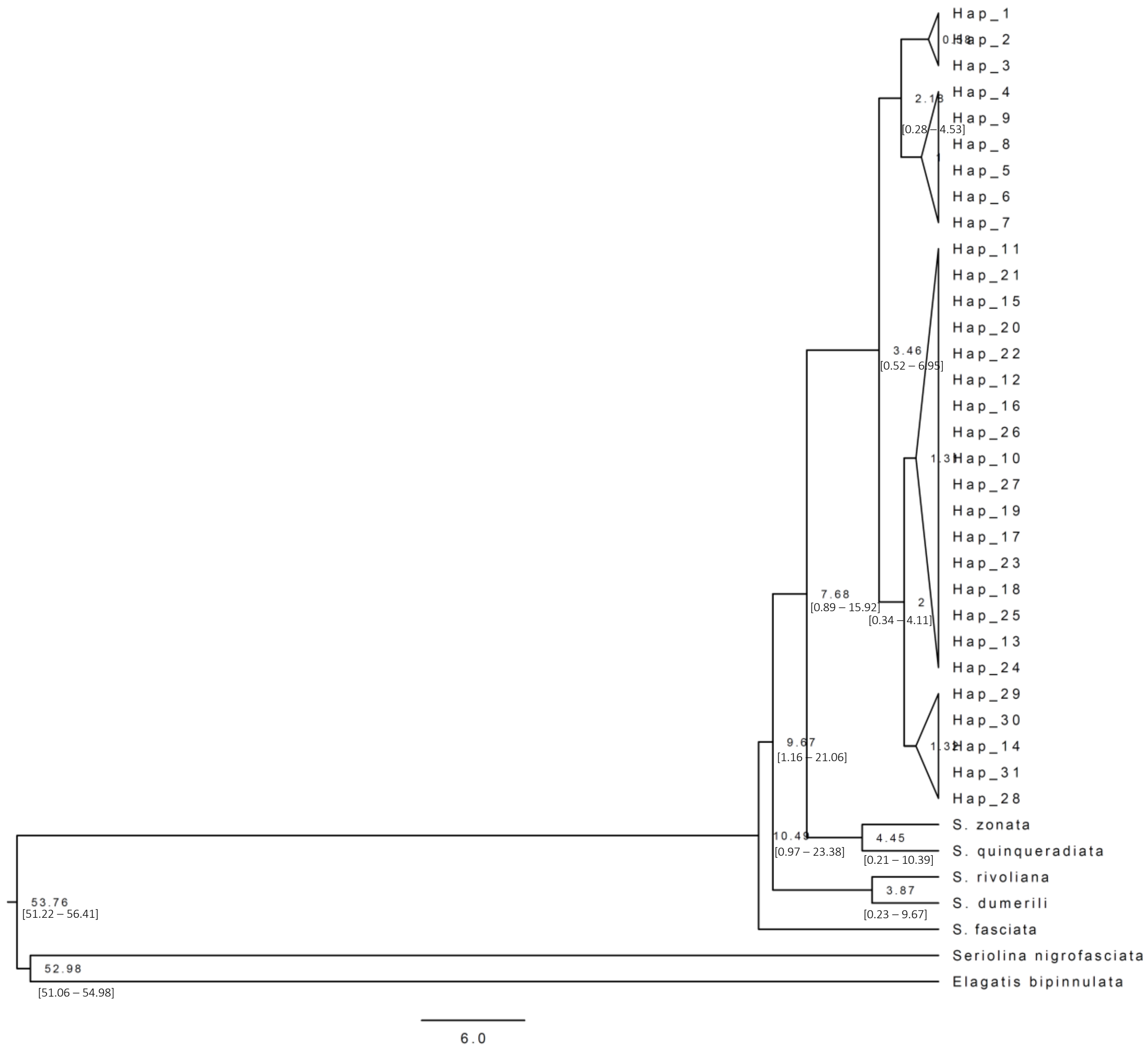

Supplementary Figure 3. Divergence times estimated for mtDNA COI (present and published data) from Bayesian inferences in BEAST. Mean divergence times are indicated next to the nodes and 95% highest posterior density values (HPD) are indicated in parenthesis. The sample locations for Hap_1 – Hap_31 are given in Supplementary Table1.

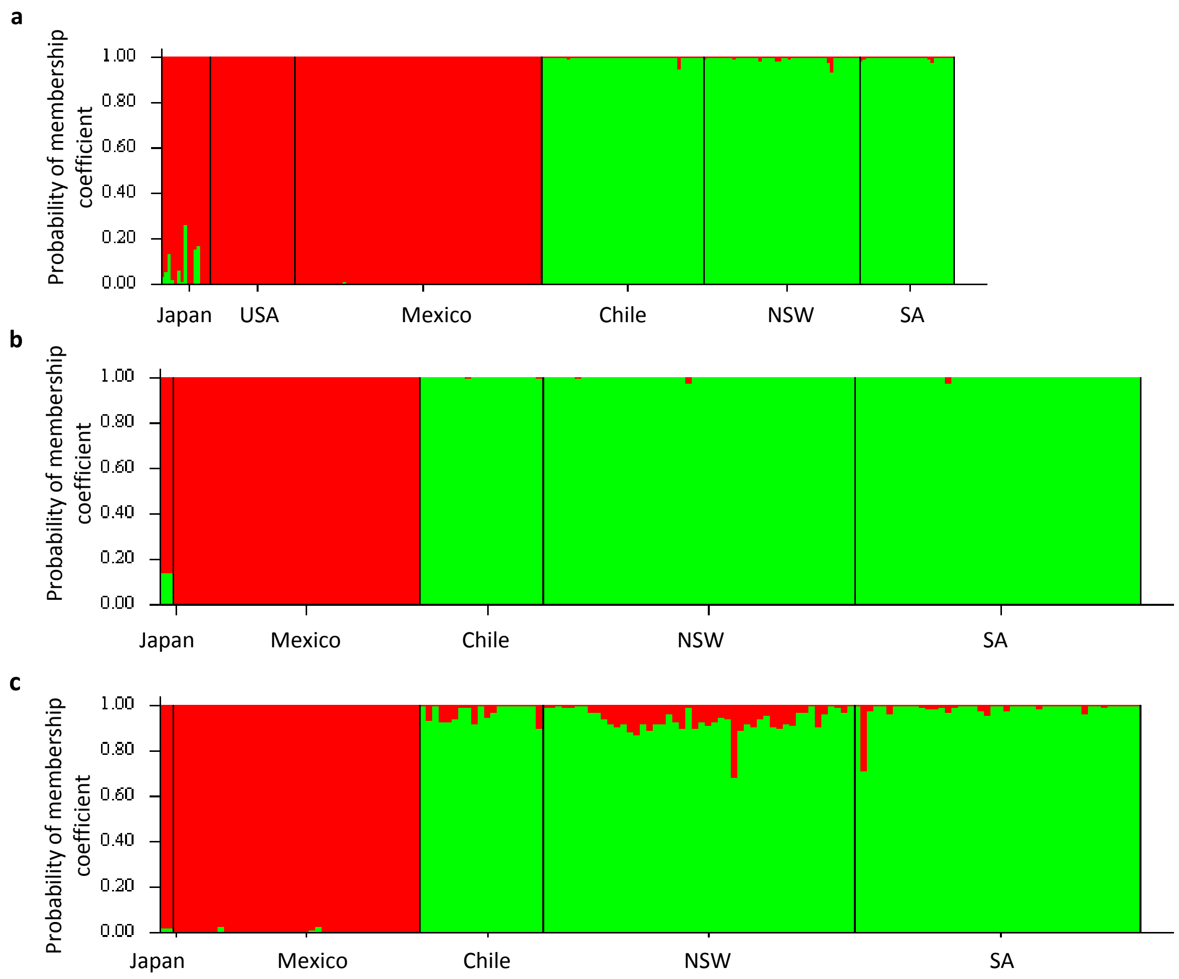

Supplementary Figure 4. Cluster analysis from STRUCTURE for a) microsatellite, b) SNPs and c) DArT markers at K = 2.

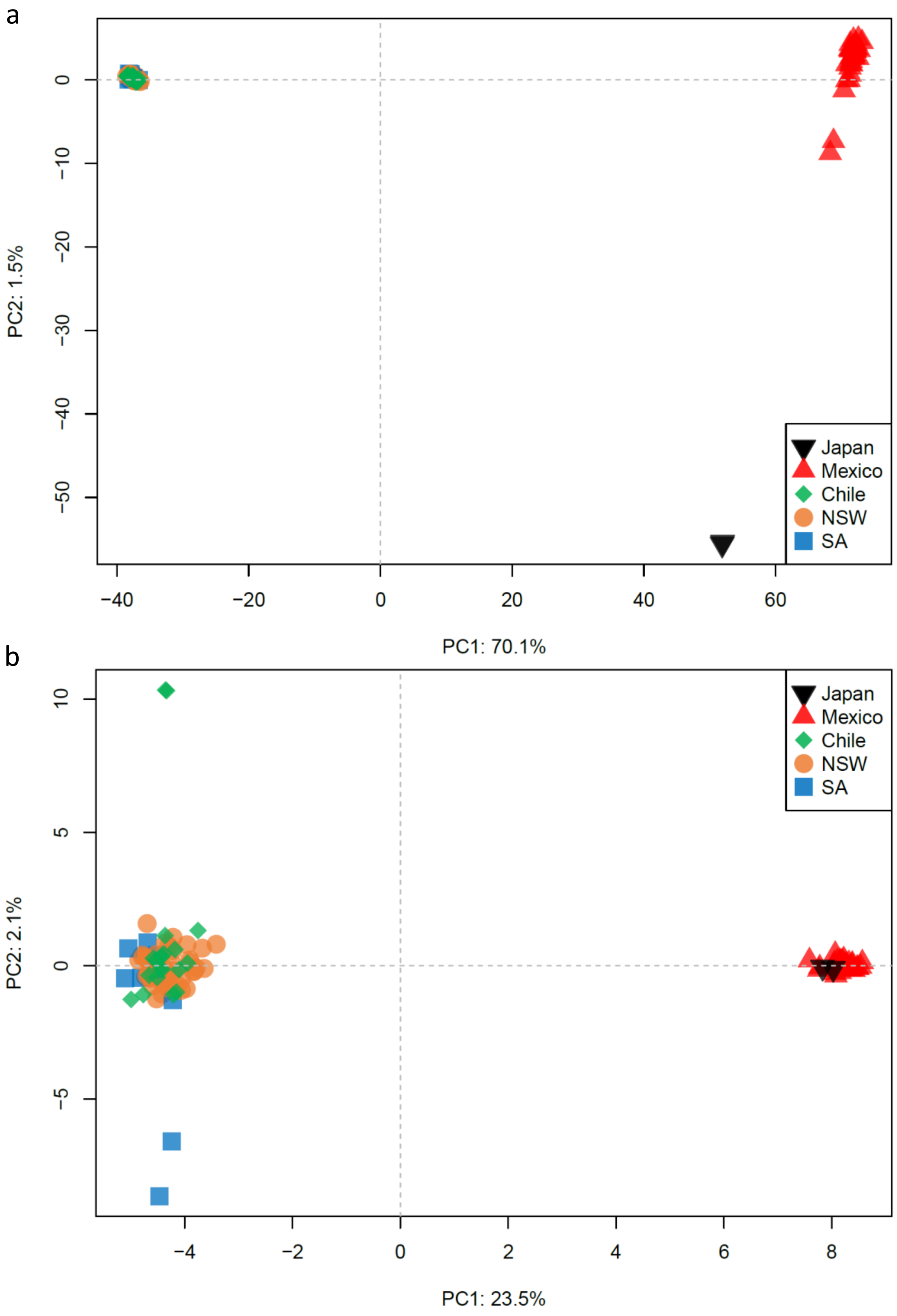

Supplementary Figure 5. Individual relationships within and between populations derived from principal component analysis (PCA) for a) SNPs and b) DArT markers.

Reference

1 Whatmore, P. *et al.* Genetic parameters for economically important traits in yellowtail kingfish *Seriola lalandi*. *Aquaculture* **400-401**, 77-84 (2013).
